# Cold-Pressed Pomegranate Seed Oil: Study of Punicic Acid Properties by Coupling of GC/FID and FTIR

**DOI:** 10.3390/molecules27185863

**Published:** 2022-09-09

**Authors:** Aleksandra Zielińska, Krzysztof Wójcicki, Dorota Klensporf-Pawlik, Marta Marzec, Massimo Lucarini, Alessandra Durazzo, Joel Fonseca, Antonello Santini, Izabela Nowak, Eliana B. Souto

**Affiliations:** 1 Institute of Human Genetics, Polish Academy of Sciences, Strzeszyńska 32, 60-479 Poznań, Poland; 2 Institute of Quality Science, Poznań University of Economics and Business, Aleje Niepodległości 10, 61-875 Poznań, Poland; 3 Faculty of Chemistry, Adam Mickiewicz University in Poznań, Uniwersytetu Poznańskiego 8, 61-614 Poznań, Poland; 4 CREA-Research Centre for Food and Nutrition, Via Ardeatina 546, 00178 Rome, Italy; 5 Department of Pharmaceutical Technology, Faculty of Pharmacy, University of Porto, Rua de Jorge Viterbo Ferreira 228, 4050-313 Porto, Portugal; 6 Department of Pharmacy, University of Napoli Federico II, Via D. Montesano 49, 80131 Napoli, Italy; 7 REQUIMTE/UCIBIO, Faculty of Pharmacy, University of Porto, Rua de Jorge Viterbo Ferreira 228, 4050-313 Porto, Portugal

**Keywords:** vegetable oils, punicic acid, pomegranate seed oil, gas chromatography, flame-ionization detection, MIR spectroscopy

## Abstract

Over the last decades, we have witnessed an increasing interest in food-related products containing vegetable oils. These oils can be obtained either by extraction or by mechanical pressing of different parts of plants (e.g., seeds, fruit, and drupels). Producers of nutraceuticals have ceaselessly searched for unique and effective natural ingredients. The enormous success of argan oil has been followed by discoveries of other interesting vegetable oils (e.g., pomegranate oil) containing several bioactives. This work describes the pomegranate fruit extract and seed oil as a rich source of conjugated linolenic acid as a metabolite of punicic acid (PA), deriving from the omega-5 family (ω-5). Through the chemical characterization of PA, its nutritional and therapeutic properties are highlighted together with the physiological properties that encourage its use in human nutrition. We analyzed the composition of all fatty acids with beneficial properties occurring in pomegranate seed oil using gas chromatography (GC) with flame-ionization detection (FID) analysis combined with Fourier transform infrared spectroscopy (FTIR). Pomegranate seed oil mainly consists of 9,11,13-octadic-trienoic acid (18:3), corresponding to 73 wt % of the total fatty acids. Nine components were identified by GC in PSO, varying between 0.58 and 73.19 wt %. Using midinfrared (MIR) spectroscopy, we compared the composition of pomegranate seed oil with that of meadowfoam seed oil (MSO), which is also becoming increasingly popular in the food industry due to its high content of long chain fatty acids (C20-22), providing increased oil stability. From the results of FTIR and MIR spectroscopy, we found that punicic acid is unique in PSO (73.19 wt %) but not in MSO.

## 1. Introduction

Vegetable oils have become commonly approved and widely used in the food market due to their particular content of fatty acids [1,2]. From the chemical point of view, these oils are a combination of glycerin with higher fatty acids of long aliphatic carbon chains (min. C14:0). Each vegetable oil shows different properties depending on the percentage of saturated and unsaturated higher fatty acids [3,4]. The presence of valuable lipids in oils leads to the generation of an occlusive film on the skin, restricting the transepidermal water loss (TEWL), which is conducive to maintaining the correct moisture content in the epidermis. Vegetable oils also protect and regenerate the stratum corneum, alleviate inflammation, and enhance the proper structure of the skin’s intercellular cement [4,5,6]. From the perspective of the nutraceutical function, researchers have investigated the relationship between vegetable oils and health benefits [7,8], as well as the improvement in the target delivery of bioactives [9,10,11,12,13,14]. Among vegetable oils, cold-pressed oils are attracting attention because, as derivatives (have undergone minimum or no processing), they are generally considered higher quality [15,16]. Many oils exhibit unique flavors, odors, and special characteristics relevant to cosmetics, therapeutics, and dietary products. They are promoted as specialty oils [17]. Due to their complex nature, the quality of the extracts also needs to be of a high standard so that their composition is compatible with the detection system [18].

An example of an emerging specialty oil is the oil obtained from pomegranate seeds in the process of cold mechanical pressing or from pomegranate fruit by CO_2_ extraction. Pomegranate fruit, flowers, and bark are often used in cosmetology [19]. Pomegranate fruits are edible and used to prepare drinks [20]. The extract from pomegranate fruit contains tannins and astringent properties and is a component of cosmetic products for dying hair [21]. These extracts longer retain the hair dyes, and the hair assumes a mild red hue. Pomegranate fruit juice improves the skin texture, and milled pomegranate seed can be used for peeling [22]. The extracts of *Punica granatum* Linn were also proposed for use in wound healing when formulated in chitosan dressings [23]. The extract from its fresh flowers is used to alleviate injuries and swellings, in addition to their use as a dye in cosmetics and fabrics [23,24]. Pomegranate (*Punica granatum*) belongs to the family *Punicicaceae* and most probably comes from India [23,25]. The pomegranate tree bark contains 0.3–0.7 wt % of piperidinium alkaloids, including peletierine, pseudopeletierine, and isopeletierine, in addition to up to 25 wt % of tannins [26]. The fruit is a berry that breaks up on maturation, initially coated with a thick red skin that changes to brown with time. The mature fruit contains many seeds (from about 200 to 1400). Each is surrounded by a water-laden pulp of refreshing sour taste that is rich in anthocyanins, vitamin C, malic acid, citric acid, oxalic acid, pectins, sugars, and mucilaginous compounds [25,27]. The juice of *Punica granatum* fruit and its seed oil show strong antioxidative properties, much higher than those of red wine and close to those of green tea. The high content of punicic acid (PA) and the richness of flavonoids are responsible for hindering the activity of prostaglandins and postinflammation enzymes, so that pomegranate seed oil (PSO) is effective for treating acne vulgaris [28,29]. When topically applied to the skin, it accelerates the keratinocytes division, thickens the epidermis, and restricts the formation of new blood vessels (angiogenesis) [23]. PSO is also used for the treatment of *Acne rosacea* and as a support in the prophylaxis of skin cancer originating from UV irradiation (photocarcinogenesis) [29,30]. The isoflavones in PSO, mainly genistein and daidzein, increase skin density and are applied in cosmetic products to increase skin firmness. The other phytoestrogens show antiwrinkling effects, as they stimulate collagen, elastin, and hyaluronic acid biosynthesis. PSO is also used to regenerate damaged epidermis and care for mature, dry, and peeling skin [24,30,31].

According to the literature, pomegranate seed oil is composed of a group of fatty acids [4,32]. The compositions of extracts from pomegranate fruit are very similar in the range of bioactives and fatty acids; the differences can be in the percentage contents of a particular compound. The extract and the oil are rich sources of rarely occurring punicic acid [33,34,35], representing conjugated fatty acids. This acid exhibits potent anti-inflammatory and antiedematous properties and high antioxidation activity [36,37]. The commercial extract in cosmetic preparations (recommended content up to 5 wt %) shows skin nutritive activity, strengthens the skin, and has antiwrinkling activity. PSO may be applied to improve nutrition and as a skin moisturizer by improving elasticity and healing damaged epidermis. In cosmetic products, it is often applied as an active ingredient in antiwrinkling or lighting preparations and irritant-alleviating creams [24,31]. This oil enhances the synthesis of collagen and contributes to the strengthening of the stratum corneum. It shows natural estrogenic, antioxidative, antibacterial, and protective properties. Owing to its anti-inflammatory properties, it alleviates swelling and pain and heals minor irritations. It can be used to treat psoriasis and eczema, effectively soothing sunburnt skin and minor skin injuries.

**Figure 1:**
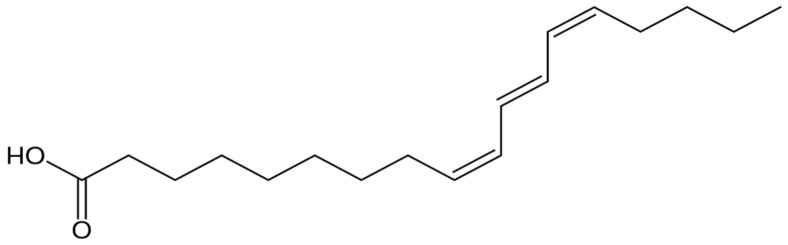
Chemical structure of punicic acid.

Based on the recent literature, the beneficial effects of punicic acid (PA, Figure 1) on human health have been confirmed. PA has the ability to reduce lipids in blood plasma and has anticancer, antioxidative, antidiabetic, and antiatherosclerotic properties [38,39,40]. Punicic acid can inhibit the proliferation of breast cancer and the growth of cancer cells [41]. A recent report indicated the significant potential for punicic acid in the prevention and treatment of prostate cancer [42]. Another report confirmed punicic acid’s chemopreventive properties toward skin cancer [42]. The review by Khajebishak et al. [38] indicated punicic acid as a potential compound of pomegranate seed oil that can be used for type 2 diabetes mellitus management.

The mechanism of PA activity has not yet been fully explained, but it is supposed that its main elements are the transformations initiated by the inhibition of biosynthesis of prostaglandins [43]. The effect of PA on diabetes has been studied; however, until now, no satisfactory decrease in the glucose concentration in the blood plasma has been obtained [44]. São Paulo University reported the potential application of PA as a functional food product. However, the effect of PA on the metabolism of lipids and weight loss has not yet been confirmed [40]. Fadavi et al. (2006) [34], however, pointed out the significant potential of PA to decrease the level of lipids in the blood and its beneficial effect on the treatment of inflammatory bowel disease [34]. Guerra-Vázquez et al. (2022) recently revised the role of PA in the treatment of neurological disorders, strongly attributing it to its antioxidant and anti-inflammatory properties [45].

Pomegranate seed oil is primarily produced by cold pressing. The product is a red oil with a characteristic fruity, solid smell. The cosmetic industry also employs CO_2_ extract a dark-yellow oil with a typical odor from pomegranate fruit. This product is obtained by supercritical carbon dioxide extraction, in which CO_2_ is passed through the plant material under high pressure. After extraction completion, CO_2_ is released, leaving pure extract. With this method, highly pure plant extracts free from chemical contaminants, such as heavy metals or residues of organic solvents, can be obtained. Currently, there are two standard methods to analyze oils. The first type of method is high-pressure liquid chromatography (HPLC), used to separate triglycerides (TGs) and identify the position of fatty acids in glycerides [46]. Białek et al. (2020) [47] reported the possibility of using the argent metric liquid chromatography to determine the quality of commercial edible pomegranate oils, including conjugated fatty acids detection; however, to obtain the profile of fatty acids, the authors used gas chromatography (GC) coupled with mass spectrometry. Therefore, if the information about TGs is not the most important result, there is a second method to analyze oil samples using GC. Gas chromatography is one of the most widely used methods for separating, identifying, and quantifying fatty acids in oils and food lipids. As a rapid, sensitive, and precise method, the obtained results show good reproducibility. Although the resolution of GC is affected by several factors, it is relatively easy to optimize. Gas chromatography methods require a transmethylation procedure to obtain the fatty acids methyl esters before the final separation. Additionally, fatty acid methyl esters can be easily detected and identified with a flame ionization detector, which is the GC detector commonly used mainly due to its superior ability to measure hydrocarbons.

Although Sassano et al. (2009) [48] reported some fundamental drawbacks of the GC analysis of conjugated fatty acids, this is still one of the most commonly used methods for fatty acids analysis [49,50]. These researchers also reported that GC analysis cannot resolve jacaric acid from punicic acid, as a significant CLA isomer in pomegranate oil [48]. To establish the potential presence of jacaric acid, Sassano et al., employed ^13^C NMR, but jacaric acid was not found in the analyzed samples. Therefore, they suggested that using GC alone for pomegranate seed oil may accurately measure punicic acid levels. In our study, we aimed to characterize pomegranate seed oil by coupling gas chromatography (GC) with flame-ionization detection (FID) and Fourier transform infrared spectroscopy (FTIR).

## 2. Materials and Methods

### 2.1. Pomegranate Seed Oil

The analyzed oil was cold-pressed pomegranate seed oil, purchased from a local market (therefore, the exact cold extraction procedure was company knowledge, not ours), which we compared with Meadowfoam Seed Oil™ (Natural Plant Products, Inc., Salem, OR, USA).

### 2.2. Fatty Acids Methyl Ester (FAME) Preparation and GC/FID Analysis

The fatty acids profile of cold-pressed pomegranate seed oil was determined using gas chromatography. However, this method requires the preparation of fatty acids as volatile methyl esters. Therefore, fatty acids methyl esters were obtained by direct methylation with a 14 wt % BF_3_-MeOH procedure. BF_3_-MeOH (Sigma-Aldrich, St. Louis, MO, USA) is one of the most convenient products used for fatty acids derivatization. It is a methanol–catalyst system that quickly and quantitatively converts fatty acids to their methyl esters when used in excess with heating. Derivatization was preceded by saponification. A lipid sample placed in a screw-capped glass tube was hydrolyzed with 1 mL of 0.5 M KOH in methanol, kept at 75 °C for 15 min, and then derivatized with 2 mL of BF_3_-MeOH then treated with n-heptane. The solution was treated with saturated NaCl solution under vigorous stirring, and an organic layer was obtained for further analysis. Fatty acids methyl esters (FAMEs) were separated on a BPX-70 capillary column (60 m × 0.25 mm × 0.25 µm; SGE Analytical Science, Munich, Germany) installed in an Agilent Technologies 7820 A gas chromatograph equipped with an automatic liquid sampler (ALS, Agilent Technologies 7693 A Santa Clara, CA, USA), and a flame-ionization detector (FID, Thermo Fisher Scientific, Waltham, MA, USA). Helium was used as the carrier gas at a flow rate of 0.8 mL/min. The column temperature was programmed from 140 °C to 240 °C at 6 °C/min. The initial and final temperatures were held for 5 and 20 min, respectively. The detector temperature was set to 270 °C. Fatty acids in oils were identified by comparing the retention times with those of authentic standards (37-component FAME mix, Supelco, Merck KGaA, Darmstadt, Germany) and the literature database [35]. The percentage content of individual components was calculated by the area normalization method. Samples were prepared in at least three independent replications.

### 2.3. Midinfrared (MIR) Spectroscopy

The mi-infrared (MIR) spectra were obtained on an FT-IR 4700 spectrometer (Jasco, Tokyo, Japan) using the attenuated total reflection (ATR) technique (ATR PRO ONE produced by Jasco). Fourier transform infrared spectroscopy (FTIR) requires minimum or no sample preparation and allows a rapid characterization of samples; indeed, it can be considered an innovative, green, and rapid methodology [51,52].

The analysis was carried out on PSO and standard cold-pressed oil, meadowfoam seed oil (MSO), was used for comparison. For each sample, the MIR spectra were recorded (32 scans per sample or background) from 4000–600 cm^−1^ at a resolution of 4 cm^−1^. The spectra were corrected using the background spectrum of air and CO_2_ reduction. The analysis was conducted at room temperature. Before acquiring a range, the ATR crystal was carefully cleaned with ethanol and acetone. The cleansed crystal was spectrally checked to ensure no residue was retained from the previous sample. For a measurement, one droplet (20 µL) of the oil was placed on the surface of the ATR crystal and covered with a glass lid to avoid contamination with ambient moisture. For each sample, ten spectra were recorded. Measurements were performed in triplicate. We qualitatively analyzed the functional groups. Spectra were analyzed concerning the spectral band positions to identify the signatures of the major functional groups. The leading bands were assigned by analyzing the acquired spectra and comparing PSO signals that represent MSO signals, considering data in the literature [35].

## 3. Results and Discussion

**Figure 2:**
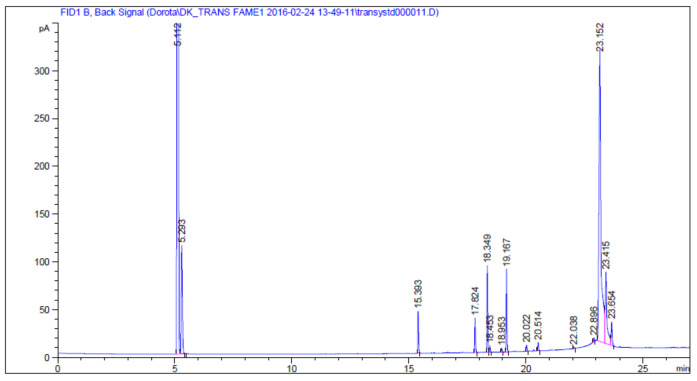
Typical GC chromatogram of the fatty acid separation of pomegranate seed oil.

A typical GC chromatogram of the fatty acid separation of pomegranate seed oil is shown in Figure 2, and the fatty acids composition of the pomegranate seed oil, obtained by GC/FID analysis, is presented in Table 1. 

We identified fatty acids methyl esters by comparing the retention times of peaks in a sample with those of commercially available standard pure compounds (Supelco 37-component FAME mix) and references [35]. The quantification of individual fatty acids was based on a relative percentage basis. Pomegranate seed oil is a specific, highly unsaturated oil. It mainly consists of 9,11,13-octadic-trienoic acid (18:3), over 73 wt % of total fatty acids, identified by comparison with previously published data [33,53]. Moreover, as much as about 70 wt % of the composition of the oil is the rarely found punicic acid (PA, up to 15 wt %) [54], which is associated with conjugated linoleic acid (CLA), containing isomers *cis*-9, *trans*-11, and *cis*-13-. These compounds are responsible for the exceptional therapeutic properties of PSO [55]. The other components of this oil are saturated fatty acids: palmitic acid (5–8 wt %) and stearic acid (2–6 wt %), unsaturated fatty acids, including linoleic acid (9–10 wt %) and oleic acid (8–9 wt %) [4]. The unsaponifiable fraction also contains phytosterols (0.4–0.6 wt %), including β-sitosterol (beta-sitosterol), stigmasterol, campesterol (up to 1 wt %), coumestans, and phytoestrogens, including isoflavones: genistein and daidzein, polyphenols: ellagic acid, or isoquercetin. Additional components of the extract are antioxidants from rosemary (*Rosmarinus officinalis*) leaf extract (0.05 wt %) [29]. Because of the content of phytoestrogens, pomegranate oil can alleviate menopause symptoms, and it is recommended for topical use as a moisturizer or as a diet supplement. Pomegranate seed oil shows strong antioxidizing properties owing to its contents of α- and γ-tocopherols. The polyphenols are derivatives of ellagic acid, flavonoids, and anthocyanins, and are responsible for their anticancer activity, antiatherosclerotic effect, and antifibrosis activity [29].

GC identified nine PSO components, and their contents varied from 0.58 to 73.19 wt % (Table 1). The three significant acids in PSO are punicic, linoleic, and oleic acids. Collectively, these three acids constitute about 88.24 wt % of the total fatty acids in pomegranate seed oil. According to the literature [56,57], the major fatty acid in PSO is punicic, accounting for 72.4–84.1 wt % of the total fatty acids, which confirms our results. A recent study also found other conjugated linolenic acids (CLnAs), different isomers in addition to punicic acids [33,34]. However, pomegranate seed oil contains a much higher amount of CLnAs than other well-known sources, such as bitter gourd (60 wt %), pot marigold (29.5 wt %), or even catalpa (27.5 wt %) [58]. Considering the overall fatty acid composition, we found that PSO contains: 83.4 wt % of polyunsaturated fatty acids (PUFA), 9.09 wt % of monounsaturated fatty acids, and 7.51 wt % of saturated fatty acids. Our results agree with those reported in the literature for qualitative and quantitative fatty acid profiles [52,53]. However, the differences in the design may be due to the effect of the cultivar type and region of cultivation [33,34].

The significance of PA is related to its high content in PSO, reaching 73 wt %. Because of its high content, this acid was assumed to be responsible for the oil’s beneficial properties [4]. This acid is rarely found and is characteristic of the pomegranate seed oil from *Punica granatum* L., after which it was named. From the chemical point of view, PA is a polyunsaturated fatty acid from the omega-5 family [4,40]. According to the International Union of Pure and Applied Chemistry (IUPAC) nomenclature, it is (Z,E,Z)-9,11,13-octadecatrienoic acid [4], which means that its molecule is composed of 18 carbon atoms and has three conjugated double bonds, at the 9th, 11th, and 13th carbon atom, counting from the carboxyl group. It is assumed that 66% of the double bonds in punicic acid are of the *cis*- type, while 33% are the *trans*- type [40]. Punicic acid is an isomer of α-linoleic acid [59]. The two compounds show many structural similarities, e.g., in the number of carbon atoms, their distribution, and the number of double bonds in the carbon chain. Punicic acid shares several properties with α-linoleic acid (the latter is an essential fatty acid), which is beneficial for the human skin [40]. The properties of PA determine that the oil acts as a cosmetic ingredient. The content of PA mainly contributes to the oil’s effect of swelling reduction, alleviation of skin irritations, anti-inflammation, and antioxidative activity. The oil acts as a natural UV filter owing to the ellagic acid content. Punicic acid has several effects that are beneficial for the skin, both epidermis and dermis. First, it restricts the excessive transepidermal water loss [4] and accelerates regenerative processes by intensifying the skin’s production of proteins and barrier lipids. This activity protects against excessive skin drying and epidermis peeling [29]. Punicic acid regulates cell division and shows a robust anti-inflammatory effect in the mechanism of inhibition of prostaglandins synthesis [29].

**Figure 3:**
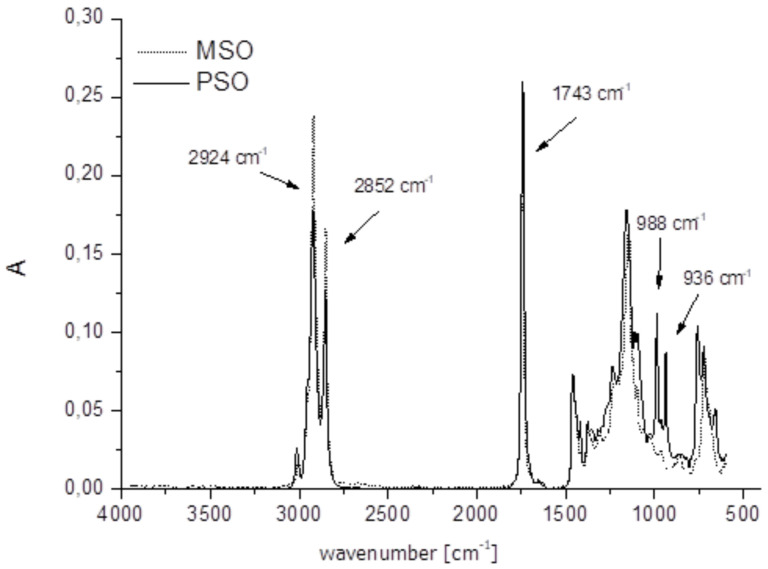
MIR spectra (4000–600 cm^−1^) of PSO and MSO. (A, absorbance).

The results of FTIR show a characteristic signature of a sample’s chemical or biochemical compounds by featuring their molecular vibrations (stretching, bending, and torsions of the chemical bonds) [60]. Therefore, the FTIR spectrum represents a molecular fingerprint of a sample. The MIR spectra of the analyzed oils are presented in Figure 3. Each peak in the FTIR spectra corresponds to the functional groups and vibration modes responsible for infrared absorption. Spectrum bands originating from triacylglycerols, the main components of oils, dominate the typical MIR spectra of vegetable oil, although other components in the oil also contribute to the spectra [61]. For better visualization and discussion, we compared PSO with other cold-pressed oil, namely meadowfoam seed oil (MSO), which is also popular in the cosmetic industry because of its composition of fatty acids, over 95 wt % of which has chain lengths of 20 carbon atoms or longer [62]. The spectral profile of PSO differs from the spectral profile of MSO. Some bands are different in shape and intensity appear due to different unsaturated and saturated fatty acid contents. The intensive bands with maxima at 2924 and 2852 cm^−1^ arise, respectively, from the asymmetric and symmetric stretching vibrations of CH_2_ methylene and terminal methyl groups of the fatty acid chains in triacylglycerols [61]. These bands are more intense for MSO than for PSO (Figure 3). The intensive band with a maximum at 1743 cm^−1^ originates from the stretching vibrations of the carbonyl group (C=O) present in the glycerol fatty acid ester bonds (COOR) of triacylglycerols [58]. The spectral range from 1700 to 500 cm^−1^ is more intensive for PSO than for MSO. The shape of its bands also significantly differs in the range of 1000–700 cm^−1^, where two intensive bands occur (with maxima at 988 and 936 cm^−1^). These bands probably correspond with punicic acid, which is only present in PSO (73.19 wt %).

The results of MIR spectroscopy showed the differences in the shape and intensity of some bands due to the different unsaturated and saturated fatty acid contents of both oils. The obtained results proved that vibrational spectroscopy methods can be successfully used for analyzing the chemical composition of natural oils, such as PSO and MSO. Moreover, using MIR spectroscopy, it was possible to identify the presence of punicic acid, which is unique in PSO.

## 4. Conclusions

The general purpose of our study was to compare PSO and MSO by MIR analysis and to confirm the content of fatty acids and other ingredients by using GC/FID. Using MIR spectroscopy, we found that the differences in the shape and intensity of some bands appear due to the different unsaturated and saturated fatty acid contents of both oils. Those differences have also been proven using GC/FID chromatography. Our study successfully showed that by using GC/FID-MIR, it is possible to identify the presence of PA, which occurs only in PSO. As expected, pomegranate seed oil is a rich source of PA, representing more than 70 wt % of the total fatty acids, and contains a significant amount of CLA, tocopherols, and sterols.

## Figures and Tables

**Table 1:** Composition of fatty acids of PSO.

Saturation of Fatty Acid	Common Name of Fatty Acid	Systematic Name of Fatty Acid	Numerical Symbol	Peak Area Percentage in Oil (mean wt % ± SD)
Saturated fatty acids	Palmitic acid	Hexadecanoic acid	C16:0	3.92 ± 0.09
	Stearic acid	Octadecanoic acid	C18:0	3.00 ± 0.07
	Arachidic acid	Eicosanoic acid	C20:0	0.59 ± 0.02
Unsaturated fatty acids	Oleic acid	*cis*-9-octadecenoic acid	C18:1; ω-9	7.69 ± 0.13
		*cis*-11-octadecenoic acid	C18:1; ω-7	0.58 ± 0.01
	Linoleic acid	(9Z,12Z)-octadeca-9,12-dienoic acid	C18:2; ω-6	7.36 ± 0.16
	Paullinic acid	(13Z)-icos-13-enoic acid	C20:1; ω-7	0.82 ± 0.06
	**Punicic acid**	**(9Z,11E,13Z)-octadecatrienoic acid**	C18:3	**73.19 ± 0.50**
			C18:3; (isomer)	2.85 ± 0.12

## Data Availability

Not applicable.

## References

[1] Alexovič M., Dotsikas Y., Bober P., Sabo J. (2018). Achievements in robotic automation of solvent extraction and related approaches for bioanalysis of pharmaceuticals. J. Chromatogr. B.

[2] Ramos-Payán M. (2019). Liquid—Phase microextraction and electromembrane extraction in millifluidic devices: A tutorial. Anal. Chim. Acta.

[3] Rotondo A., La Torre G.L., Dugo G., Cicero N., Santini A., Salvo A. (2020). Oleic Acid Is not the Only Relevant Mono-Unsaturated Fatty Ester in Olive Oil. Foods.

[4] Zielińska A., Nowak I. (2014). Fatty acids in vegetable oils and their importance in cosmetic industry. CHEMIK.

[5] Alves A.Q., da Silva V.A., Góes A.J.S., Silva M.S., de Oliveira G.G., Bastos I., de Castro Neto A.G., Alves A.J. (2019). The Fatty Acid Composition of Vegetable Oils and Their Potential Use in Wound Care. Adv. Skin Wound Care.

[6] Vaughn A.R., Clark A.K., Sivamani R.K., Shi V.Y. (2018). Natural oils for skin-barrier repair: Ancient compounds now backed by modern science. Am. J. Clin. Dermatol..

[7] Blanco-Llamero C., Fonseca J., Durazzo A., Lucarini M., Santini A., Señoráns F.J., Souto E.B. (2022). Nutraceuticals and Food-Grade Lipid Nanoparticles: From Natural Sources to a Circular Bioeconomy Approach. Foods.

[8] Campos J.R., Severino P., Ferreira C.S., Zielinska A., Santini A., Souto S.B., Souto E.B. (2019). Linseed Essential Oil—Source of Lipids as Active Ingredients for Pharmaceuticals and Nutraceuticals. Curr. Med. Chem..

[9] Carbone C., Martins-Gomes C., Caddeo C., Silva A.M., Musumeci T., Pignatello R., Puglisi G., Souto E.B. (2018). Mediterranean essential oils as precious matrix components and active ingredients of lipid nanoparticles. Int. J. Pharm..

[10] Carbone C., Teixeira M.D.C., Sousa M.D.C., Martins-Gomes C., Silva A.M., Souto E.M.B., Musumeci T. (2019). Clotrimazole-Loaded Mediterranean Essential Oils NLC: A Synergic Treatment of Candida Skin Infections. Pharmaceutics.

[11] Zielińska A., Ferreira N.R., Feliczak-Guzik A., Nowak I., Souto E.B. (2020). Loading, release profile and accelerated stability assessment of monoterpenes-loaded Solid Lipid Nanoparticles (SLN). Pharm. Dev. Technol..

[12] Zielinska A., Martins-Gomes C., Ferreira N.R., Silva A.M., Nowak I., Souto E.B. (2018). Anti-inflammatory and anti-cancer activity of citral: Optimization of citral-loaded solid lipid nanoparticles (SLN) using experimental factorial design and LUMiSizer(R). Int. J. Pharm..

[13] Souto E.B., Zielinska A., Souto S.B., Durazzo A., Lucarini M., Santini A., Silva A.M., Atanasov A.G., Marques C., Andrade L.N. (2020). (+)-Limonene 1,2-epoxide-loaded SLNs: Evaluation of drug release, antioxidant activity and cytotoxicity in HaCaT cell line. Int. J. Mol. Sci..

[14] Vieira R., Severino P., Nalone L.A., Souto S.B., Silva A.M., Lucarini M., Durazzo A., Santini A., Souto E.B. (2020). Sucupira Oil-Loaded Nanostructured Lipid Carriers (NLC): Lipid Screening, Factorial Design, Release Profile, and Cytotoxicity. Molecules.

[15] Celenk V., Gumus Z.P., Ustun Argon Z., Buyukhelvacigil M., Karasulu E. (2018). Analysis of Chemical Compositions of 15 Different Cold pressed Oils Produced in Turkey: A Case Study of Tocopherol and Fatty Acid Analysis. J. Turk. Chem. Soc. Sect. A Chem..

[16] Fine F., Brochet C., Gaud M., Carre P., Simon N., Ramli F., Joffre F. (2016). Micronutrients in vegetable oils: The impact of crushing and refining processes on vitamins and antioxidants in sunflower, rapeseed, and soybean oils. Eur. J. Lipid Sci. Technol..

[17] Hernandez E.M., Sanders T.A.B. (2016). Specialty Oils: Functional and Nutraceutical Properties. Functional Dietary Lipids: Food Formulation, Consumer Issues and Innovation for Health.

[18] Zhang Q.-W., Lin L.-G., Ye W.-C. (2018). Techniques for extraction and isolation of natural products: A comprehensive review. Chin. Med..

[19] Liu G., Xu X., Hao Q., Gao Y. (2009). Supercritical CO_2_ extraction optimization of pomegranate (*Punica granatum* L.) seed oil using response surface methodology. LWT.

[20] Mohagheghi M., Rezaei K., Labbafi M., Ebrahimzadeh Mousavi S.M. (2011). Pomegranate seed oil as a functional ingredient in beverages. Eur. J. Lipid Sci. Technol..

[21] Sumit K., Vivek S., Sujata S., Ashish B. (2012). Herbal cosmetics: Used for skin and hair. Inventi J..

[22] Derakhshan Z., Ferrante M., Tadi M., Ansari F., Heydari A., Hosseini M.S., Conti G.O., Sadrabad E.K. (2018). Antioxidant activity and total phenolic content of ethanolic extract of pomegranate peels, juice and seeds. Food Chem. Toxicol..

[23] do Nascimento M.F., Cardoso J.C., Santos T.S., Tavares L.A., Pashirova T.N., Severino P., Souto E.B., Albuquerque-Junior R.L.C. (2020). Development and Characterization of Biointeractive Gelatin Wound Dressing Based on Extract of *Punica granatum* Linn. Pharmaceutics.

[24] Jurenka J. (2008). Therapeutic applications of pomegranate (*Punica granatum* L.): A review. Altern. Med. Rev..

[25] Mahesar S.A., Kori A.H., Sherazi S.T.H., Kandhro A.A., Laghari Z.H. (2019). Pomegranate (*Punica granatum*) Seed Oil. Fruit Oils: Chemistry and Functionality.

[26] Jayaprakash A. (2017). *Punica granatum*: A Review on Phytochemicals, Antioxidant and Antimicrobial Properties. J. Acad. Ind. Res..

[27] Yahia E.M. (2011). Postharvest Biology and Technology of Tropical and Subtropical Fruits: Fundamental Issues.

[28] Wang R., Wang W., Wang L., Liu R., Ding Y., Du L. (2006). Constituents of the flowers of *Punica granatum*. Fitoterapia.

[29] Grossmann M.E., Mizuno N.K., Schuster T., Cleary M.P. (2010). Punicic acid is an ω-5 fatty acid capable of inhibiting breast cancer proliferation. Int. J. Oncol..

[30] Carvalho Filho J.M. (2014). Pomegranate seed oil (*Punica granatum* L.): A source of punicic acid (conjugated α-linolenic acid). J. Hum. Nutr. Food Sci..

[31] Aslam M.N., Lansky E.P., Varani J. (2006). Pomegranate as a cosmeceutical source: Pomegranate fractions promote proliferation and procollagen synthesis and inhibit matrix metalloproteinase-1 production in human skin cells. J. Ethnopharmacol..

[32] Natalello A., Hervás G., Toral P.G., Luciano G., Valenti B., Mendoza A.G., Pauselli M., Priolo A., Frutos P. (2020). Bioactive compounds from pomegranate by-products increase the in vitro ruminal accumulation of potentially health promoting fatty acids. Anim. Feed Sci. Technol..

[33] Eikani M.H., Golmohammad F., Homami S.S. (2012). Extraction of pomegranate (*Punica granatum* L.) seed oil using superheated hexane. Food Bioprod. Process..

[34] Fadavi A., Barzegar M., Azizi M.H. (2006). Determination of fatty acids and total lipid content in oilseed of 25 pomegranates varieties grown in Iran. J. Food Compos. Anal..

[35] Jing P., Ye T., Shi H., Sheng Y., Slavin M., Gao B., Liu L., Yu L.L. (2012). Antioxidant properties and phytochemical composition of China-grown pomegranate seeds. Food Chem..

[36] Baradaran Rahimi V., Ghadiri M., Ramezani M., Askari V.R. (2020). Anti-inflammatory and anti-cancer activities of pomegranate and its constituent, ellagic acid: Evidence from cellular, animal, and clinical studies. Phytother. Res..

[37] Moghadam E.H., Shaaban M., Sepahvand A. (2020). Medicinal Properties of Pomegranate. Herb. Med. J..

[38] Khajebishak Y., Payahoo L., Alivand M., Alipour B. (2019). Punicic acid: A potential compound of pomegranate seed oil in Type 2 diabetes mellitus management. J. Cell. Physiol..

[39] Huber R., Gminski R., Tang T., Weinert T., Schulz S., Linke-Cordes M., Martin I., Fischer H. (2017). Pomegranate (*Punica granatum*) Seed Oil for Treating Menopausal Symptoms: An Individually Controlled Cohort Study. Altern. Ther. Health Med..

[40] Saha S., Ghosh M. (2009). Comparative study of antioxidant activity of α-eleostearic acid and punicic acid against oxidative stress generated by sodium arsenite. Food Chem. Toxicol..

[41] Wang L., Martins-Green M. (2014). Pomegranate and its components as alternative treatment for prostate cancer. Int. J. Mol. Sci..

[42] Hora J.J., Maydew E.R., Lansky E.P., Dwivedi C. (2003). Chemopreventive effects of pomegranate seed oil on skin tumor development in CD_1_ mice. J. Med. Food..

[43] Nekooeian A.A., Eftekhari M.H., Adibi S., Rajaeifard A. (2014). Effects of pomegranate seed oil on insulin release in rats with type 2 diabetes. Iran. J. Med. Sci..

[44] Viladomiu M., Hontecillas R., Lu P., Bassaganya-Riera J. (2013). Preventive and prophylactic mechanisms of action of pomegranate bioactive constituents. Evid. Based Complement. Alternat. Med..

[45] Guerra-Vázquez C.M., Martínez-Ávila M., Guajardo-Flores D., Antunes-Ricardo M. (2022). Punicic Acid and Its Role in the Prevention of Neurological Disorders: A Review. Foods.

[46] Van Nguyen A., Deineka V., Deineka L., Vu Thi Ngoc A. (2017). Comparison of separation of seed oil triglycerides containing isomeric conjugated octadecatrienoic acid moieties by reversed-phase HPLC. Separations.

[47] Białek A., Białek M., Lepionka T., Tober E., Czauderna M. (2021). The Quality Determination of Selected Commercial Online Purchased Edible Pomegranate Seed Oils With New Argentometric Liquid Chromatography Method. J. Diet. Suppl..

[48] Sassano G., Sanderson P., Franx J., Groot P., van Straalen J., Bassaganya-Riera J. (2009). Analysis of pomegranate seed oil for the presence of jacaric acid. J. Sci. Food Agric..

[49] Amri Z., Lazreg-Aref H., Mekni M., El-Gharbi S., Dabbaghi O., Mechri B., Hammami M. (2017). Oil characterization and lipids class composition of pomegranate seeds. BioMed Res. Int..

[50] Alfekaik D.F., AL-Hilfi S.A. (2016). Fatty Acids Composition by (GC-MS) and Most Important Physical Chemicals Parameters of Seed Oil Pomegranate and Grape Seeds. J. Biol. Agric. Healthc..

[51] Lucarini M., Durazzo A., Kiefer J., Santini A., Lombardi-Boccia G., Souto E.B., Romani A., Lampe A., Ferrari Nicoli S., Gabrielli P. (2019). Grape Seeds: Chromatographic Profile of Fatty Acids and Phenolic Compounds and Qualitative Analysis by FTIR-ATR Spectroscopy. Foods.

[52] Mahesar S.A., Lucarini M., Durazzo A., Santini A., Lampe A.I., Kiefer J. (2019). Application of Infrared Spectroscopy for Functional Compounds Evaluation in Olive Oil: A Current Snapshot. J. Spectrosc..

[53] Fernandes L., Pereira J.A., Lopéz-Cortés I., Salazar D.M., Ramalhosa E., Casal S. (2015). Lipid composition of seed oils of different pomegranate (*Punica granatum* L.) cultivars from Spain. Int. J. Food Stud..

[54] Boroushaki M.T., Mollazadeh H., Afshari A.R. (2016). Pomegranate seed oil: A comprehensive review on its therapeutic effects. Int. J. Pharm. Sci. Res..

[55] Puneeth H., Chandra S. (2020). A review on potential therapeutic properties of Pomegranate (*Punica granatum* L.). Plant Sci. Today.

[56] Melgarejo P., Artes F. (2000). Total lipid content and fatty acid composition of oilseed from lesser known sweet pomegranate clones. J. Sci. Food Agric..

[57] Özgül-Yücel S. (2005). Determination of conjugated linolenic acid content of selected oil seeds grown in Turkey. J. Am. Oil Chem. Soc..

[58] Wójcicki K., Khmelinskii I., Sikorski M., Caponio F., Paradiso V.M., Summo C., Pasqualone A., Sikorska E. (2015). Spectroscopic techniques and chemometrics in analysis of blends of extra virgin with refined and mild deodorized olive oils. Eur. J. Lipid Sci. Technol..

[59] Viladomiu M., Hontecillas R., Yuan L., Lu P., Bassaganya-Riera J. (2013). Nutritional protective mechanisms against gut inflammation. J. Nutr. Biochem..

[60] Yap K.Y.-L., Chan S.Y., Lim C.S. (2007). Infrared-based protocol for the identification and categorization of ginseng and its products. Food Res. Int..

[61] Vlachos N., Skopelitis Y., Psaroudaki M., Konstantinidou V., Chatzilazarou A., Tegou E. (2006). Applications of Fourier transform-infrared spectroscopy to edible oils. Anal. Chim. Acta.

[62] Zielińska A., Dąbrowska M., Nowak I. (2015). Olej z nasion meadowfoam—“Perła” wśród olejów roślinnych. Pol. J. Cosmetol..

